# The complete chloroplast genome of *Gynura japonica* and its phylogenetic implications

**DOI:** 10.1080/23802359.2022.2102443

**Published:** 2022-07-29

**Authors:** Mimi Li, Xin Xian, Bingru Ren, Hongjiang Wang, Weilin Li, Jian Chen

**Affiliations:** a Institute of Botany, Jiangsu Province and Chinese Academy of Sciences, Nanjing, China; b The Jiangsu Provincial Platform for Conservation and Utilization of Agricultural Germplasm, Nanjing, China; c Co-Innovation Center for Sustainable Forestry in Southern China, Forestry College, Nanjing Forestry University, Nanjing, China

**Keywords:** Asteraceae, chloroplast genome, *Gynura japonica*, Senecoineae

## Abstract

*Gynura japonica* (Asteraceae) is a folk herbal medicine with multi-pharmacological functions involving analgesic, hemostatic and antiangiogenic activities. The study was conducted to assemble the complete chloroplast (cp) genome of *G. japonica* through a genome-skimming approach. The assembled cp genome was 151,023 bp in size, with 62.8% AT content, consisting of a large single copy (LSC) of 83,185 bp, two copies of inverted repeat (IRa and IRb) of 24,847 bp, and a small single copy (SSC) of 18,144 bp. The cp genome of *G. japonica* contained 133 genes, including eight ribosomal RNA genes (rRNAs), 37 transfer RNA genes (tRNAs), 86 protein-coding genes (PCGs), and two pseudogenes (ψ*ycf*1 and ψ*rps*19). Our phylogenomic analysis based on whole plastid genomes strongly supports *G. japonica* is a sister to the clade including *Crassocephalum crepidioides* and *Jacobaea vulgaris*.

The genus *Gynura* Cass. is a small genus of the tribe Senecoineae, family Asteraceae. It consists of approximately 40 species distributed through tropical Africa to Asia with one species in Australia (Chen and Nordenstam 2011). *Gynura japonica* (Thunberg) Juel 1891 is widely located in tropical Asia, China, Japan, Nepal, and Thailand. It has been used as a folk herbal medicine with various pharmacological fuctions involving analgesic, hemostatic and antiangiogenic activities (Li et al. 2015). Although considerable studies have been conducted on *Gynura* species (Vanijajiva and Kadereit 2011), the taxonomy of *Gynura* has remained poorly understood. Previous works traditionally relied on morphological characteristics, which may result in ambiguities in species delimitation (Hebert et al. 2003). Fortunately, whole cp genome sequences can provide useful information for taxonomic treatment. The study was performed to obtain the whole cp genome of *G*. *japonica*, which was valuable for species identification and taxonomic treatment of genus *Gynura*.

*Gynura japonica* was cultivated and collected in Nanjing Botanical Garden, Memorial Sun Yat-Sen, China (32°3′32″N, 118°49′50″E). Sampling permissions were obtained from the Institute of Botany, Jiangsu Province and Chinese Academy of Sciences, China. A specimen was preserved at the Department of Medical Plants, Institute of Botany, Jiangsu Province and Chinese Academy of Sciences (http://cnbg.net/science/herb, Jian Chen and E-mail: chenjian80@aliyun.com) under voucher number 2018S47. Genomic DNA (gDNA) was extracted through a modified CTAB (cetyltrimethylammonium bromide) method (Doyle and Doyle 1987). The concentration and quality of gDNA were determined using a NanoDrop™ 2000 spectrophotometer (Thermo Scientific, Waltham, USA). The DNA library was prepared with an Illumina library prep kit and subsequently sequenced on an Illumina HiSeq paired-end (PE) sequencing platform (San Diego, USA) at Novogene (Beijing, China). The software NOVOPlasty 4.3.1 was employed to directly assemble the raw reads into plastid genome (Dierckxsens et al. 2017) with default setting and using *Senecio vulgaris* (NC046693) as a reference sequence. Gene annotations were performed by GeSeq (Tillich et al. 2017) and adjusted start/stop codons manually in Geneious 11.1.5 (Kearse et al. 2012). The newly generated cp sequence has been submitted to the GenBank of NCBI (https://www.ncbi.nlm.nih.gov/) with accession number MZ935743.

The whole cp genome of *G. japonica* was 151,023 bp in a quadripartite double-stranded structure, including an LSC (large single copy) of 83,185 bp, a pair of IRs (inverted repeats) of 24,847 bp and an SSC (small single copy) of 18,144 bp. The GC content was 37.2%, of which was 35.3% in LSC, 30.3% in SSC, and 42.9% in IRs regions, respectively. There were 133 genes were annotated, which consisted of eight ribosomal RNA genes (rRNA), 37 transfer RNA genes (tRNA), 86 protein-coding genes (PCGs), and two pseudogenes (ψycf1 and ψrps19). Twenty genes contained one or two introns.

Whole-plastome alignments of 25 Senecioneae were conducted using MAFFT 7.409 (Katoh and Standley 2013) implemented in Geneious. The phylogenomic analysis was performed by maximum likelihood (ML) in RAxMLv8.2 (Stamatakis 2014) under GTR + GAMMA model with 1000 bootstrap replicates using *Petasites japonicus* (MN385243) as an outgroup. *Gynura cusimbua* (NC056914) was not added to the analysis because of possible misidentification according to Han et al. (2019). The topology of our phylogenomic analyses is strongly congruent with earlier studies based on nuclear regions and plastid fragments (Pelser et al. 2010; Fu et al. 2016) with *G. japonica* closely related to a clade including *Crassocephalum crepidioides* (MW362305) and *Jacobaea vulgaris* (NC015543) (Figure 1). Furthermore, *Senecio* was found to be a polyphyletic group, which is consistent with the results reported previously by Pelser et al. (2007; 2010).

**Figure 1.**
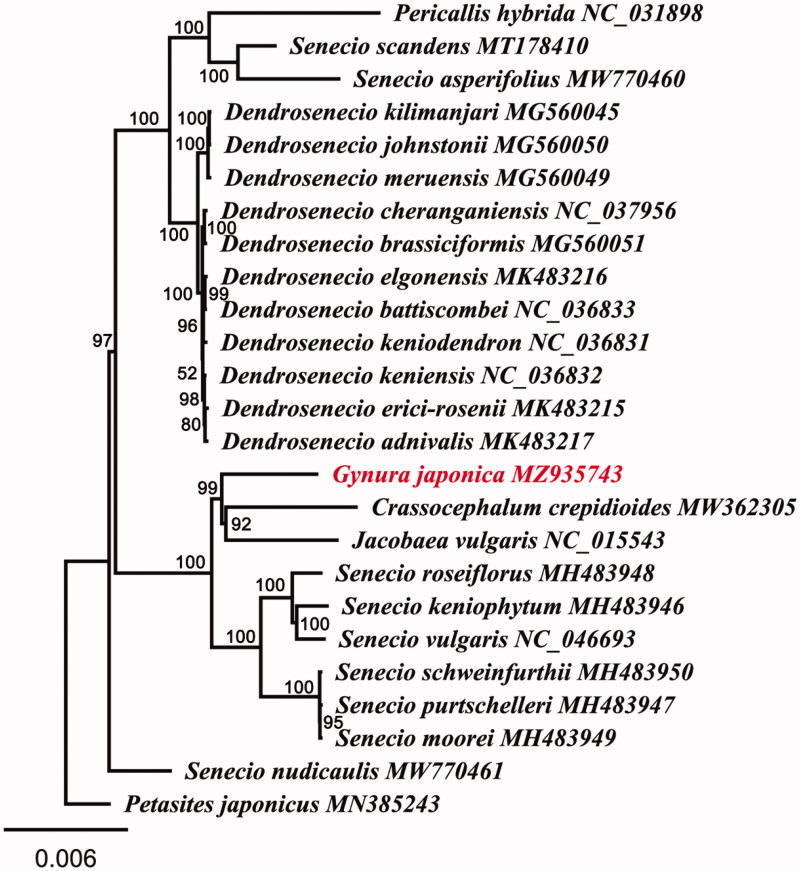
The phylogenomic tree constructed by maximum likelihood (ML) methods based on the whole chloroplast genomes. Numbers near each node are bootstrap support values.

## Data Availability

The genome sequence data that support the findings of this study are openly available in GenBank of NCBI at (https://www.ncbi.nlm.nih.gov/) under the accession no. MZ935743. The associated BioProject, SRA, and Bio-Sample numbers are PRJNA758200, SRR15647819 and SAMN21015383, respectively.
